# A barnavirus sequence mined from a transcriptome of the Antarctic pearlwort *Colobanthus quitensis*

**DOI:** 10.1007/s00705-018-3794-x

**Published:** 2018-03-07

**Authors:** Max L. Nibert, Austin R. Manny, Humberto J. Debat, Andrew E. Firth, Laura Bertini, Carla Caruso

**Affiliations:** 1 Department of Microbiology and Immunobiology, Harvard Medical School, Boston, MA 02115 USA; 2 Ph.D. Program in Virology, Harvard University, Cambridge, MA 02138 USA; 3 Instituto de Patología Vegetal, Centro de Investigaciones Agropecuarias, Instituto Nacional de Tecnología Agropecuaria (IPAVE-CIAP-INTA), X5020ICA Córdoba, Argentina; 4 Department of Pathology, Division of Virology, University of Cambridge, Cambridge, CB2 1QP UK; 5 Department of Ecological and Biological Sciences, Universita degli Studi della Tuscia, 01100 Viterbo, Italy

## Abstract

Because so few viruses in the family *Barnaviridae* have been reported, we searched for more of them in public sequence databases. Here, we report the complete coding sequence of Colobanthus quitensis associated barnavirus 1, mined from a transcriptome of the Antarctic pearlwort *Colobanthus quitensis*. The 4.2-kb plus-strand sequence of this virus encompasses four main open reading frames (ORFs), as expected for barnaviruses, including ORFs for a protease-containing polyprotein, an RNA-dependent RNA polymerase whose translation appears to rely on − 1 ribosomal frameshifting, and a capsid protein that is likely to be translated from a subgenomic RNA. The possible derivation of this virus from a fungus associated with *C. quitensis* is discussed.

**Electronic supplementary material:**

The online version of this article (10.1007/s00705-018-3794-x) contains supplementary material, which is available to authorized users.

The family *Barnaviridae* is currently represented by one species, *Mushroom bacilliform virus*, in genus *Barnavirus* [16]. The originally reported sequence for mushroom bacilliform virus (MBV) (GenBank U07551.1; also NC_001633.1) is from an Australian strain of the cultivated button mushroom (basidiomycete) *Agaricus bisporus* [19]. A closely related sequence for MBV (97% nt sequence identity) (GenBank KY357511.1) has been reported recently from a second strain of *A. bisporus* [6]. In addition, the sequence of another apparent member of the genus *Barnavirus*, Rhizoctonia solani barnavirus 1 (RsBV1; GenBank KP900904.2), has been reported recently from the phytopathogenic basidiomycete *Rhizoctonia solani* [12] but has not yet been recognized by the ICTV as a member of a separate species.

MBV has a positive-sense RNA genome that is ~ 4.0 kb long [6, 19, 20]. The genome encompasses four main ORFs (ORF1–ORF4), respectively encoding a protein of unknown function (P1), a polyprotein that includes protease and VPg domains (P2), an RNA-dependent RNA polymerase (RdRp; P3 region), and a coat/capsid protein (CP; P4) [6, 17–20] (Fig. 1). ORF3 partially overlaps ORF2 in the −1 frame and appears to be expressed as a P2+3 fusion polyprotein dependent on programmed ribosomal frameshifting [19]. ORF4 does not overlap ORF3 and is expressed instead from a subgenomic RNA containing only ORF4 [17, 19]. The ~ 3.9-kb genome of RsBV1 exhibits a coding organization closely related to that of MBV [12] (Fig. 1; the presence of ORF1 in RsBV1 is newly noted here). Based on similar genome sizes, coding organizations, and P2- and P3-region sequence similarities, both MBV and RsBV1 appear to be most closely related to plant viruses in the unassigned genus *Sobemovirus* [12, 16].

**Fig. 1:**
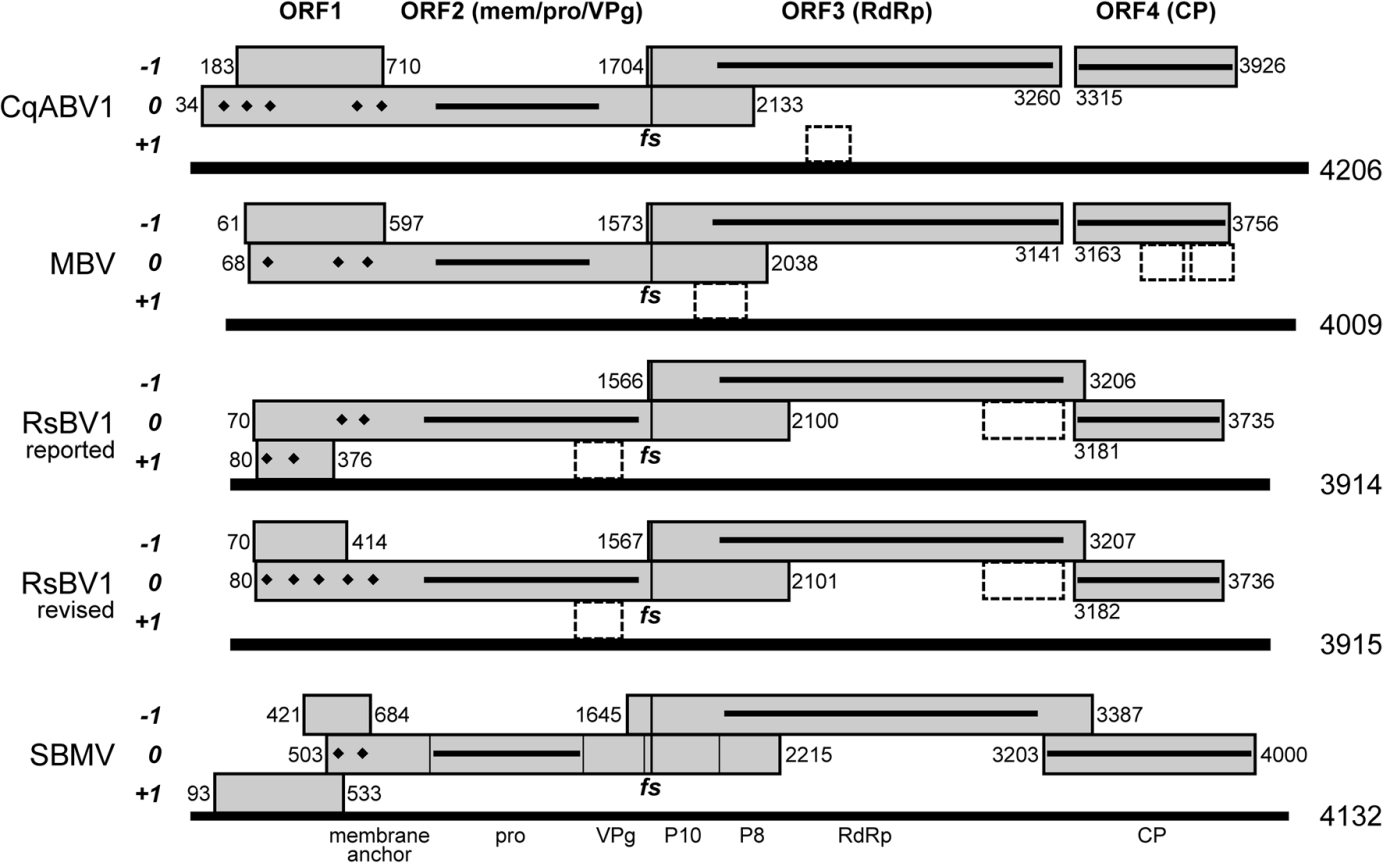
Genome organizations. Diagrams are drawn to scale and aligned to the proposed frameshifting (*fs*) site in each. For RsBV1, diagrams for both reported and newly revised sequences are included. Main ORFs are shown as gray rectangles, along with first and last nt positions (not including stop codons). The reading frame that includes ORF2 is defined as frame 0 in each genome. ORF3 is defined by bracketing stop codons; the other ORFs are defined as spanning from the first in-frame AUG codon to the first downstream stop codon. In MBV, three small ORFs with potential to encode proteins of 51–65 aa each [19] are shown as rectangles with dashed outlines; a few similarly sized small ORFs, with potential to encode proteins 52–100 aa each, are also found in CqABV1 and RsBV1 but are not located in the same genomic positions and are considered unlikely to be expressed. TM regions in encoded proteins, as predicted by SOSUI, are shown as diamonds. Regions of similarity in encoded proteins to viral proteases (pro), RdRps, or CPs, as determined by HHpred, are shown as thick lines within the respective ORFs. Known cleavage products of the ORF2-encoded polyprotein of representative sobemovirus SBMV are labeled. The ORF spanning nt 421–684 in SBMV encodes the essential sobemovirus protein Px [11], which may be analogous to barnavirus P1 based on the coding organizations of these viruses. The other 5′-proximal ORF in SBMV, spanning nt 93–533, has been reported to encode a movement protein (P1 [3]), which is not expected to be present in the fungal barnaviruses

Sobemoviruses have isometric nonenveloped virions with regular T = 3 icosahedral symmetry and a diameter of 25–30 nm [21]. MBV, in contrast, has nonenveloped virions that exhibit “bacilliform” morphology, with typical dimensions of 19 × 50 nm [20] and probable T = 1 icosahedral symmetry at the virion ends [16]. The name barnavirus reflects this bacilliform shape. This type of structure is unusual, but not unique; for example, bacilliform particles with probable T = 1 symmetry are also formed by members of the unassigned genus *Ourmiavirus* [15].

Because the sequences of so few barnaviruses had been reported to date, we decided to search for more of them in public databases. In particular for this report, we did a TBLASTN search of the Transcriptome Shotgun Assembly (TSA) database at GenBank, using the deduced P2+3 fusion polyprotein sequence of MBV as query. These efforts yielded one hit with a strongly significant E-value, 2e−115, suggesting that it represents a novel barnavirus. When this hit sequence (GenBank GCIB01019590.1) was in turn used as query for a BLASTX search of the complete non-redundant protein sequences (nr) database at GenBank, the top two hits were to MBV and RsBV1 (E-values 2e−113, identities 44 and 46%), supporting the preceding suggestion. Notably, this apparent new barnavirus sequence derives from the transcriptome of a plant, specifically from leaves of the Antarctic pearlwort *Colobanthus quitensis* (Kunth) Bartl (BioProject PRJNA2683010) [8].

Based on the length (3296 nt) and coding organization of GCIB01019590.1, relative to those of MBV, we concluded that this apparent new barnavirus sequence is complete or nearly complete at its positive-sense 3´ end, but truncated within ORF2 at its 5´ end. Importantly, the sequence reads from this transcriptome study are available as experiment SRX814890 [8] in the NCBI Sequence Read Archive (SRA). We therefore used the terminal sequences of GCIB01019590.1 as queries for MegaBLAST searches of SRX814890 in an effort to obtain a more complete sequence for the apparent new barnavirus. In this manner, and in subsequent searches that progressively extended the sequence termini, we identified two reads that added 31 nt to the positive-sense 3′ sequence of GCIB01019590.1 and, more significantly, 180 reads that combined to add 879 nt to the 5′ sequence. As a result, the consensus sequence for the apparent new barnavirus reported here (GenBank MG686618) is 4206 nt long, appears to be coding complete for ORFs 1–4 (Fig. 1), and was newly assembled from a total of 821 individual reads (reads per position: mean, 19; range, 2–35). We henceforth refer to this virus as “Colobanthus quitensis associated barnavirus 1” (CqABV1).

ORF2 and ORF3 are the two longer ORFs in CqABV1, as is also the case in MBV and RsBV1 (Fig. 1). In CqABV1, their region of overlap between stop codons spans 430 nt (positions 1704–2133), with ORF3 in the −1 frame relative to ORF2. A putative slippery sequence for −1 programmed ribosomal frameshifting is located within this region of overlap (see below) and is proposed to allow ORF3 to be expressed as part of a P2+3 fusion polyprotein. The deduced lengths of P2 and P2+3 are 700 aa and 1076 aa, respectively. P2 and the P2 region of P2+3 are notable for a region spanning aa positions 290–480 with strong similarity to viral serine proteases (top *P* value, 99.4% from HHpred analysis with defaults at https://toolkit.tuebingen.mpg.de/#/tools/hhpred [1]). The P3 region of P2+3, on the other hand, is notable for a region spanning approximately aa positions 650–1070 with very strong similarity to viral RdRps (top *P* value, 100% from HHpred). Results comparable to these were obtained for the respective MBV and RsBV1 proteins.

ORF1 and ORF4 are the two shorter ORFs in CqABV1 (Fig. 1). P4 is notable for weak similarity to viral CPs across its length (top *P* value, 95.0% from HHpred), whereas P1 appears to lack significant similarity to other proteins (top *P* value, 37.1% from HHpred). Results comparable to these were again obtained for MBV and RsBV1 (including for the revised RsBV1 P1 sequence described below). There are no in-frame stop codons 5′ to the proposed AUG start codon of CqABV1 ORF1, and it is therefore possible that its ORF1 translation initiates further upstream, either at a non-AUG start codon or at an upstream AUG if the current sequence remains 5′-incomplete. ORF2, however, cannot have an upstream initiation site due to the presence of an in-frame stop codon just 3 codons 5′ to its proposed AUG start codon.

The positive-sense sequence of MBV has been previously noted to encompass three smaller ORFs (ORF5–ORF7) in addition to the four longer ORFs described above [16, 19]. Whether these smaller ORFs are expressed remains open to question. They are not conserved at similar positions in CqABV1 or RsBV1 (Fig. 1), suggesting to us that they are probably not expressed.

In the case of southern bean mosaic virus (SBMV) and other sobemoviruses, an N-terminal portion of the ORF2-encoded polyprotein (called P2 here) is annotated in GenBank (e.g., NC_004060.2) as having membrane anchor function. Using SOSUI for online transmembrane (TM) prediction (http://harrier.nagahama-i-bio.ac.jp/sosui/sosui_submit.html [9]), we found that SBMV P2 indeed has two TM regions predicted near its N-terminus. Applying this analysis to CqABV1 P2 and MBV P2, we obtained similar results: five and three TM regions, respectively, were predicted N-terminal to the protease homology region in each (Fig. 1). Similar results were also obtained for RsBV1, but in its case, a downstream cluster of two TM regions was predicted in P2, whereas an upstream cluster of two TM regions was predicted instead in P1. No TM regions were predicted in CqABV1 P1 or MBV P1. Based on these findings, we predicted that the reported RsBV1 sequence contains an assembly error within its region of ORF1/ORF2 overlap, which has caused the 5′-terminal portions of these ORFs to be artificially swapped. Indeed, by accessing available SRA data for RsBV1 (experiment SRX1747281) and then reassembling the RsBV1 contig, we discovered that a single nt residue had been clearly omitted between nt positions 359 and 360 of the RsBV1 sequence reported in GenBank KP900904.2 (Fig. S1), causing the 5´-terminal portions of ORF1 and ORF2 to be swapped as we had predicted. By incorporating this missing residue back into the RsBV1 sequence, not only was ORF1 now shifted into the −1 frame relative to ORF2, as found in CqABV1 and MBV, but also five TM regions were now predicted via SOSUI in the N-terminal region of RsBV1 P2, vs. none in P1 (Fig. 1). To our knowledge, membrane association by N-terminal portions of the barnavirus P2 polyprotein has not been suggested previously. These membrane-associating portions of P2 seem likely to be involved in forming the RNA replication compartments of barnaviruses, as known for many other positive-sense RNA viruses [7].

The slippery sequence for −1 programmed ribosomal frameshifting in the ORF2/ORF3 overlap region of MBV has been proposed to be (G)_GUU_UUU_C, where underlines indicate codon boundaries for ORF2 and the parenthetical G was not initially included [19]. However, the canonical motif for a −1 frameshift site is X_XXY_YYZ [4], where Z is any nucleotide except G, YYY is AAA or UUU, and XXX is normally any three identical nucleotides, though a number of exceptions—including GGU—also occur [2]. Thus, the proposed slippery sequence in MBV would have been better identified as G_GUU_UUU, i.e., nudged upstream by one nt residue. Notably, this G_GUU_UUU sequence in MBV is also found at a similar position in RsBV1, and the related sequence G_AUU_UUU is found not only at a similar position in CqABV1 but also in some arteriviruses as a variant of the (normally G_GUU_UUU) nsp2N/nsp2TF −1/−2 frameshift site [2]. Given this degree of conservation (Fig. 2), we have adopted this putative slippery sequence for deducing the P2+3 fusion polyprotein sequence of CqABV1. The revised consensus motif for the barnavirus slippery sequence is thus G_RUU_UUU.

**Fig. 2:**
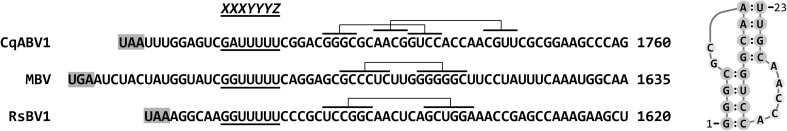
Signals for −1 programmed ribosomal frameshifting. The stop codon defining the maximal possible 5′ extent of ORF3 in each virus is shaded. The proposed −1 slippery sequence in each is underlined. Stems in the RNA structures predicted directly downstream of the slippery sequence in each are overlined; interactions between the half stems are indicated by brackets. The predicted two-stem structure in CqABV1 is a compact pseudoknot. The nt position of the 3′-most residue shown for each virus is indicated at right. A folding diagram of the proposed CqABV1 pseudoknot is shown at far right (generated using PseudoViewer 3.0 as implemented at http://pseudoviewer.inha.ac.kr/)

For efficient −1 frameshifting, a suitable slippery sequence is normally followed by an RNA structure (stem–loop or pseudoknot), separated from the shift site by a 5- to 9-nt spacer sequence [2]. A predicted long stem–loop has been identified in MBV, but beginning only 3 nt downstream [19]. This predicted stem–loop, however, includes a bulged residue near the middle of the stem, and the portion of the stem–loop after this residue is separated from the slippery sequence by 7 nt. Similarly, RsBV1 has a predicted stem–loop separated from the slippery sequence by 5 nt, and CqABV1 has a predicted compact pseudoknot also separated from the slippery sequence by 5 nt. These additional findings (Fig. 2) increase our confidence that the site for −1 programmed ribosomal frameshifting has been properly identified for these viruses.

The preceding observations suggest that CqABV1 would be properly classified with MBV and RsBV1 in family *Barnaviridae*. To address this further, we performed multiple sequence alignments and phylogenetic comparisons. Maximum-likelihood trees for P2, the P3 (RdRp) region of P2+3, or P4 showed CqABV1, MBV, and RsBV1 clustering apart from the sobemoviruses, which form their own distinct cluster (Figs. 3 and S2). These findings are also largely reflected in global pairwise comparison (Needle) scores for these proteins, although the degree of sequence conservation between CqABV1 and either MBV or RsBV1 appears fairly low in this type of analysis, especially for proteins P1, P2 and P4 (Table S1). Local pairwise comparisons with BLASTP, on the other hand, yield E-value scores that more clearly indicate the significant sequence similarities between the proteins of CqABV1 and those of MBV and/or RsBV1 (Table S2). We conclude from these results that it seems warranted at present to classify all three of these viruses in the family *Barnaviridae*, genus *Barnavirus*.

**Fig. 3:**
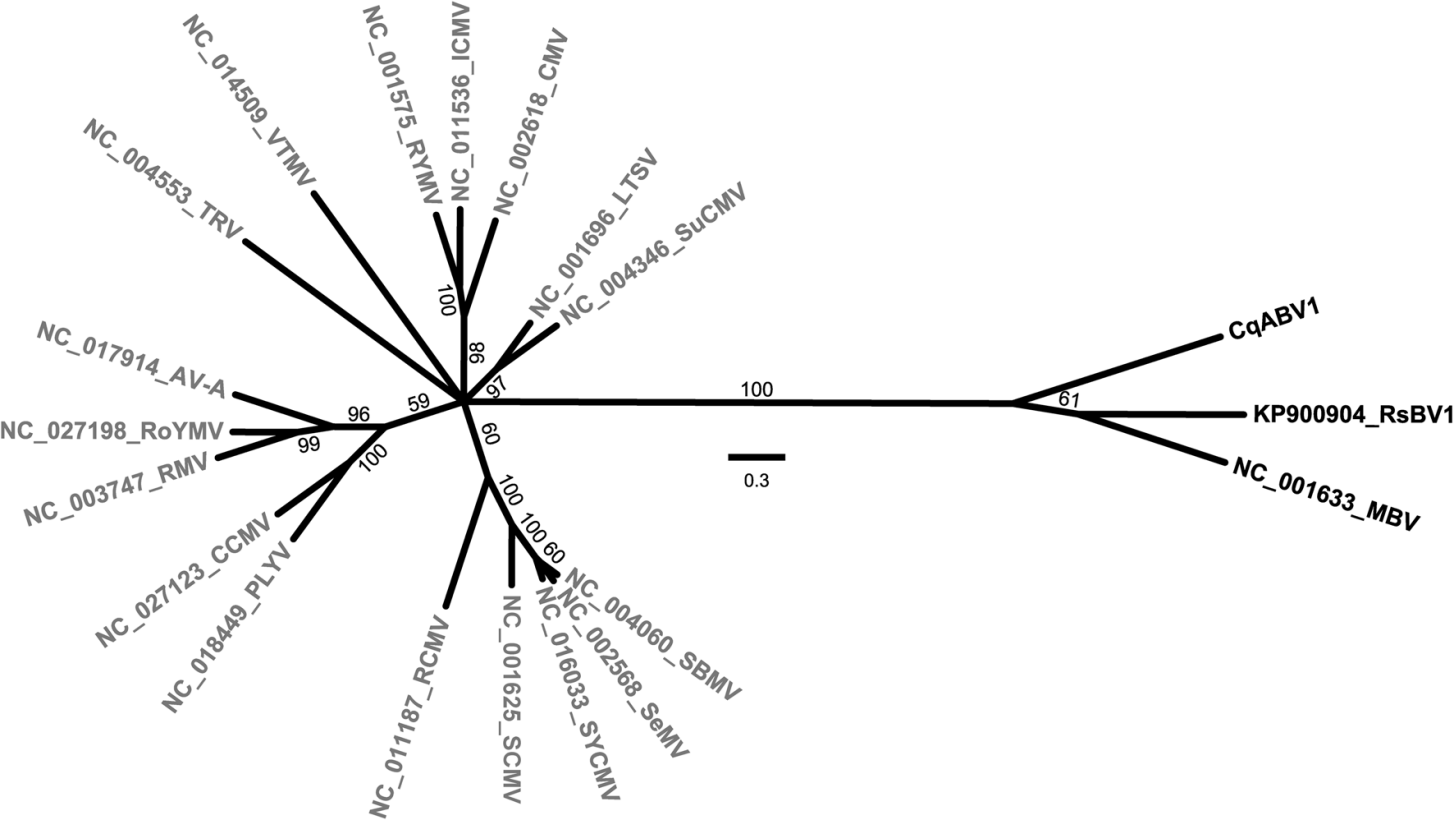
Unrooted radial phylogram. Deduced protein sequences for the P3 (RdRp) region of P2+3 of barnaviruses (black) and sobemoviruses (gray) were aligned using MAFFT 7.3 (G-INS-i) and then subjected to maximum-likelihood phylogenetic analyses using ModelFinder, IQ-TREE, and UFBoot [10, 13, 14] as implemented with the “Find best and apply” option at https://www.hiv.lanl.gov/content/sequence/IQTREE/iqtree.html. The following were found to apply: best-fit model according to BIC, LG+F+I+G4; model of rate heterogeneity, Invar+Gamma with 4 categories; proportion of invariable sites, 0.0743; and gamma shape alpha, 0.8970. Branch support values (from 1000 bootstrap replicates) are shown in %; branches with < 50% support have been collapsed to the preceding node. The scale bar indicates the average number of substitutions per alignment position. See Table S4 for a summary of abbreviations and GenBank numbers

There appears to be some question as to the specific origin of CqABV1. Within experiment SRX814890, the sequence reads are reported under six different runs, which were in turn derived from six distinct sets of sampled leaves from a mixture of individual plants. Only three of these six runs contain nearly all of the CqABV1-matching reads (Table S3), suggesting that—even within the samples from BioProject PRJNA268301—CqABV1 was largely or completely absent from some samples. Individual reads from a second transcriptome study of Antarctic pearlwort, BioProject PRJNA388703, are also available in the SRA, under experiments SRX2913822 and SRX2913823 (TSA data from this study were not yet available at the time of this report). When we used the CqABV1 genome sequence as query for MegaBLAST searches of SRX2913822 and SRX2913823, no significant hits were found (all E-values > 10). Thus, in this second study, CqABV1 appears to have been absent from all samples. One explanation for this variability in the presence of CqABV1 is that some of the sampled plants were infected with CqABV1, whereas others were not. Alternatively, the CqABV1-positive samples might have included a symbiont or contaminant infected with CqABV1, whereas the CqABV1-negative samples did not. The possibility that CqABV1 was not derived directly from Antarctic pearlwort but instead from an associated fungus, is especially intriguing, since both of the other barnaviruses reported to date, MBV and RsBV1, have fungal origins.

As a test of whether the BioProject PRJNA268301 transcriptome might include a noteworthy fraction of fungal sequences, we performed a locally run DIAMOND search [5] to try to identify the top hit (parameter –top 0) for each of the 165,386 contigs in this transcriptome. The results of this search were that 111,182 of the contigs registered a top hit with E-value ≤ 1e−05, for 79,553 (72%) of which the top hit was from a plant (kingdom Viridiplantae), consistent with this being a plant transcriptome. For 25,766 (23%), however, the top hit was instead from a fungus (kingdom Fungi), with 19,569 (18%) and 6018 (5.4%) from dikaryan phyla *Ascomycota* and *Basidiomycota*, respectively. In turn, the ascomycete hits were mostly from the classes *Leotiomycetes* (13,693; 12%), *Dothideomycetes* (2723; 2.5%), *Eurotiomycetes* (1011; 0.9%), and *Sordariomycetes* (908; 0.8%), and the basidiomycete hits were mostly from the classes *Agaricomycetes* (4989; 4.5%) and *Tremellomycetes* (867; 0.8%). A locally run BLASTN search yielded similar results, with 117,919 of the contigs registering a top hit with E-value ≤ 1e−05, for 22,624 (19%) of which the top hit was from a dikaryan fungus. These results provide evidence that the PRJNA268301 transcriptome indeed contains a noteworthy fraction of various fungal sequences, apparently representing a mixture of different dikaryan species. We thus speculate that CqABV1 was derived from one of these associated fungi, and not directly from Antarctic pearlwort. This explains our inclusion of the word “associated” in the name for this new barnavirus. Even more broadly, these findings indicate that the PRJNA268301 transcriptome, like many others reported to NCBI and elsewhere, is probably better identified as a metatranscriptome because it was derived from samples comprising more than its single, primary target organism.

## Electronic supplementary material

Below is the link to the electronic supplementary material.

Supplementary material 1 (TIFF 1281 kb)

Supplementary material 2 (TIFF 337 kb)

Supplementary material 3 (PDF 76 kb)
