# Supplementary figures and images for: A barnavirus sequence mined from a transcriptome of the Antarctic pearlwort Colobanthus quitensis
Source: Arch Virol. 2018 Mar 7;163(7):1921–6. doi: 10.1007/s00705-018-3794-x (PMC5999160; doi:10.1007/s00705-018-3794-x)

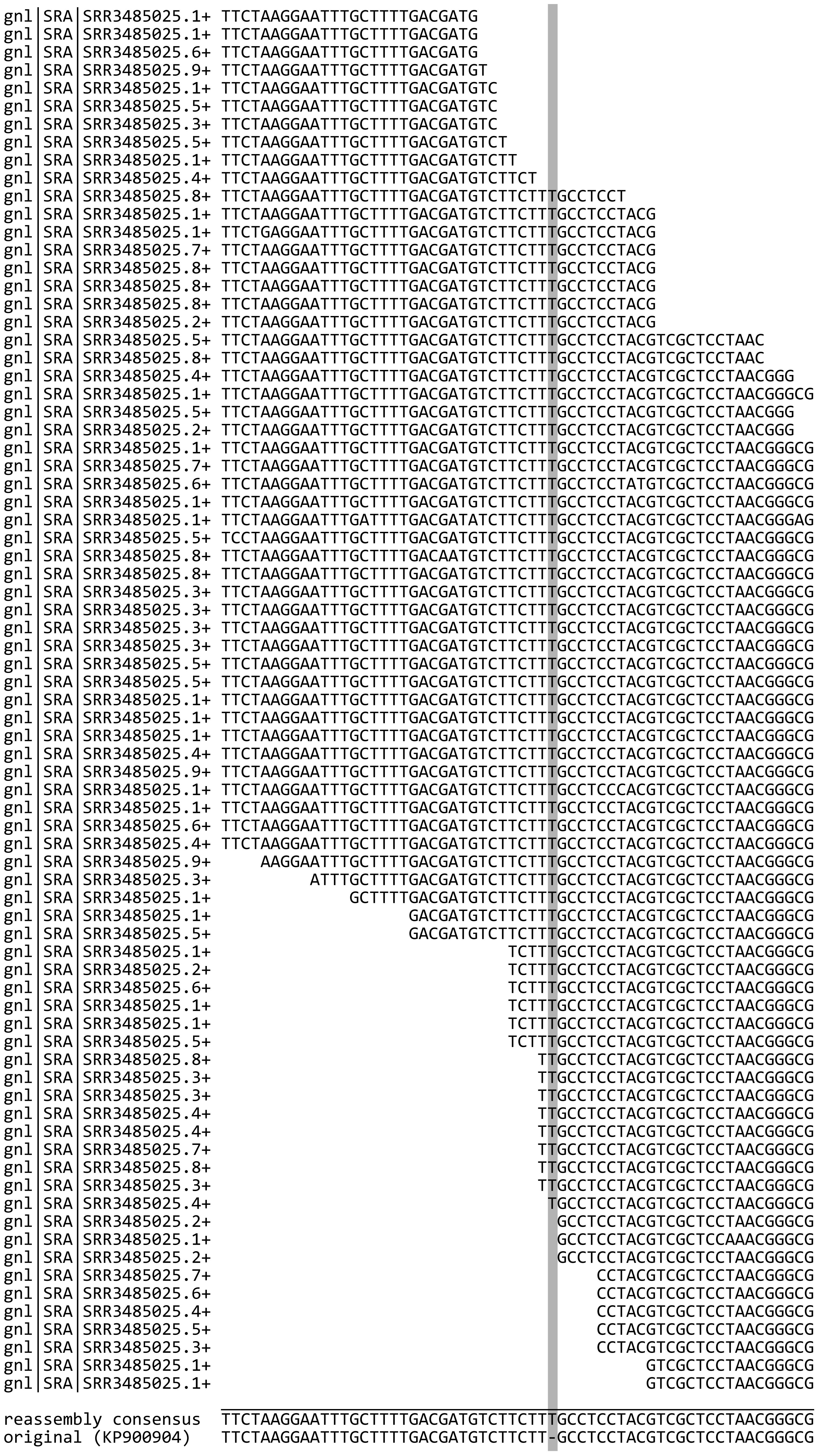

Supplement: Supplementary file 1 — Supplementary material 1 (TIFF 1281 kb) [file 705_2018_3794_MOESM1_ESM.tif]

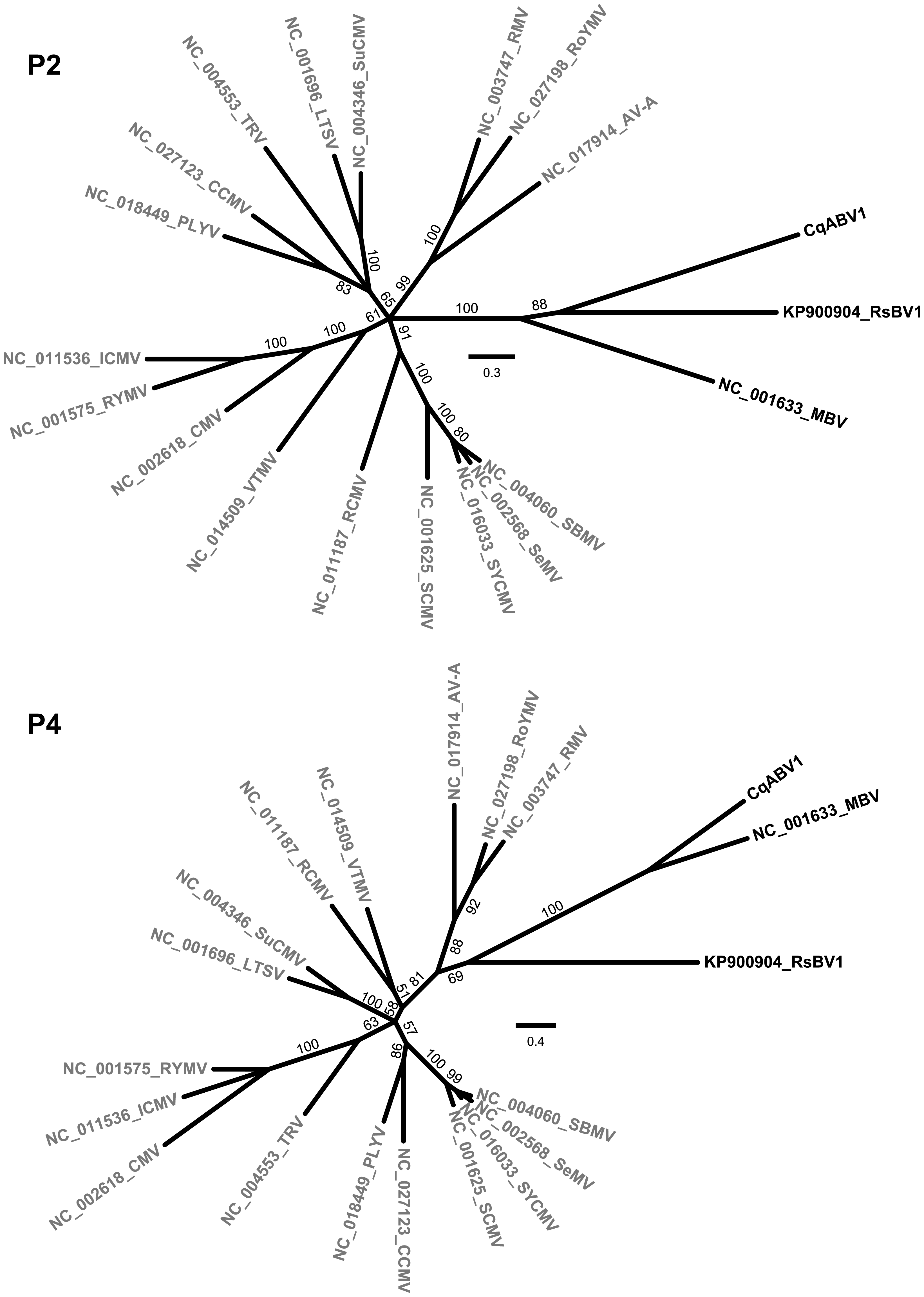

Supplement: Supplementary file 2 — Supplementary material 2 (TIFF 337 kb) [file 705_2018_3794_MOESM2_ESM.tif]
