# Supplementary material for: A barnavirus sequence mined from a transcriptome of the Antarctic pearlwort Colobanthus quitensis
Source: Arch Virol. 2018 Mar 7;163(7):1921–6. doi: 10.1007/s00705-018-3794-x (PMC5999160; doi:10.1007/s00705-018-3794-x)
Supplement: Supplementary file 3 — Supplementary material 3 (PDF 76 kb) [file 705_2018_3794_MOESM3_ESM.pdf]

Supplementary Figure Legends

Fig. S1. Alignment of sequence reads across the newly identified 1-nt insertion in RsBV1. All individual reads from experiment SRX1747281 that contributed to the new RsBV1 contig reassembly across this inserted residue are shown. The position of the inserted residue is highlighted with gray shading. The 3′-most nt residue shown here represents nt position 386 in the RsBV1 reassembly and nt position 385 in the RsBV1 assembly reported in GenBank KP900904.2.

Fig. S2. Unrooted radial phylograms. Deduced protein sequences for P2 and P4 of barnaviruses (black) and sobemoviruses (gray) were aligned using MAFFT 7.3 (G-INS-i) and subjected to maximum-likelihood phylogenetic analyses using ModelFinder, IQ-TREE, and UFBoot [10, 13, 14] as implemented with the "Find best and apply" option at https://www.hiv.lanl.gov/content/sequence/IQTREE/iqtree.html. For P2 (generally called P2a in the sobemovirus literature), the following were found to apply: best-fit model according to BIC, VT+F+R4; model of rate heterogeneity, FreeRate with 4 categories; and site proportion and rates, (0.0404,0.0575), (0.2094,0.3629), (0.4764,0.9027), and (0.2738,1.7958). For P4, the following were found to apply: best-fit model according to BIC, LG+F+I+G4; model of rate heterogeneity, Invar+Gamma with 4 categories; proportion of invariable sites, 0.0062; and gamma shape alpha, 3.2407. Branch support values (from 1000 bootstrap replicates) are shown in %; branches with <50% support have been collapsed. Scale bar indicates average number of substitutions per alignment position. See Table S4 for summary of abbreviations and GenBank numbers. The newly revised RsBV1 P2 sequence was used for this analysis.

Table S1. Global identity scores from pairwise comparisons with Needle for CqABV1 proteins

Protein	MBV[a]	RsBV1	SBMV
P1	12.6[b]	10.0[c]	13.7
P2	18.4	20.6[c]	13.8
P2+3	29.4	30.6[c]	19.5
P3[d]	41.7	39.1	23.2
P4	28.8	15.9	15.0

[a] See text for abbreviations
[b] Percentage values calculated using Needleall (http://www.bioinformatics.nl/cgi-bin/emboss/needleall)
[c] Using the newly revised P1 and P2 sequences of RsBV1
[d] The P3 region of P2+P3 only, i.e., the portion of P2+P3 that follows the ribosomal frameshift

Table S2. E-value scores from local pairwise comparisons with BLASTP for CqABV1 proteins

Protein	MBV[a]	RsBV1	SBMV
P1	7.2[b]	0.006[c]	0.33
P2	6e−17	3e−17[c]	2e−4
P2+3	7e−144	2e−139[c]	3e−49
P3[d]	2e−132	1e−130	2e−54
P4	2e−26	1.0	>10

[a] See text for abbreviations
[b] E-values calculated using the "Align two or more sequences" option in BLASTP
[c] Using the newly revised P1 and P2 sequences of RsBV1
[d] The P3 region of P2+P3 only, i.e., the portion of P2+P3 that follows the ribosomal frameshift

Table S3. Individual sequence reads for CqABV1 from experiment SRX814890

Run[a]	Sequence reads: Total	CqABV1[b]
SRR1720758	34,826,094	226
SRR1720760	43,526,022	4
SRR1720762	55,354,950	286
SRR1720763	43,892,866	1
SRR1720765	71,284,430	0
SRR1720767	62,620,342	305

[a] Each run represents a sample derived from a mixture of leaves from several different individual plants.
[b] Matching reads were identified by MEGABLAST using CqABV1 nt query.

Table S4. Abbreviations and RefSeq or GenBank accession numbers used for phylogenetic trees

Virus name	Abbrev.	Accession no.
Mushroom bacilliform virus	MBV	NC_001633.1
Rhizoctonia solani barnavirus 1	RsBV1	KP900904.2[a]
Artemisia virus A	AV-A	NC_017914.1
Cocksfoot mottle virus	CMV	NC_002618.2
Cymbidium chlorotic mosaic virus	CCMV	NC_027123.1
Imperata yellow mottle virus	ICMV	NC_011536.1
Lucerne transient streak virus	LTSV	NC_001696.2
Papaya lethal yellowing virus	PLYV	NC_018449.1
Rice yellow mottle virus	RYMV	NC_001575.2
Rottboellia yellow mottle virus	RoYMV	NC_027198.1
Rubus chlorotic mottle virus	RCMV	NC_011187.1
Ryegrass mottle virus	RMV	NC_003747.2
Sesbania mosaic virus	SeMV	NC_002568.2
Southern bean mosaic virus	SBMV	NC_004060.2
Southern cowpea mosaic virus	SCMV	NC_001625.2
Soybean yellow common mosaic virus	SYCMV	NC_016033.1
Subterranean clover mottle virus	SuCMV	NC_004346.1
Turnip rosette virus	TRV	NC_004553.3
Velvet tobacco mottle virus	VTMV	NC_014509.2

[a] The newly revised RsBV1 P2 sequence was used.
